# Perceived ideal number of children among adolescent girls in sub-Saharan Africa: does exposure to family planning messages matter?

**DOI:** 10.1186/s12905-023-02609-4

**Published:** 2023-09-09

**Authors:** Million Phiri, Musonda Lemba, Liness Shasha, Milika Sikaluzwe, Simona Simona

**Affiliations:** 1 Department of Population Studies, School of Humanities and Social Sciences, University of Zambia, Lusaka, Zambia; 2 Demography and Population Studies Programme, Schools of Public Health and Social Sciences, University of the Witwatersrand, Johannesburg, South Africa; 3 Department of Social Work and Sociology, School of Humanities and Social Sciences, University of Zambia, Lusaka, Zambia

**Keywords:** Fertility preference, Adolescents, Family planning, Sub-Saharan Africa

## Abstract

**Background:**

Even though evidence shows that fertility transition has begun almost everywhere in sub–Saharan Africa (SSA), the decline has been slower than in other parts of the world. Research shows that there is a positive relationship between fertility levels and fertility preference. Therefore, many countries in the region are implementing family planning education campaigns targeting at influencing reproductive behavior of women. Thus, this study aimed to examine the extent to which exposure to family planning communication influences fertility preferences of adolescent girls in SSA.

**Methods:**

This study used data extracted from the most recent Demographic and Health Survey datasets for 28 countries in SSA. Analyses were conducted on a pooled sample of 87,950 female adolescents’ aged 15–19 years who were captured in respective country’s survey. Multivariable binary logistic regression model was fitted in Stata version 17 software to examine the association between exposure to family planning communication and fertility preference among adolescent girls in SSA.

**Results:**

The average fertility preference among adolescent girls in SSA was 4.6 children (95% CI: 4.5, 4.7). Findings show that regardless of the country, adolescents who had exposure to family planning messages [aOR = 0.76, 95% CI = 0.72–0.80] were less likely to prefer 4 or more children. On average, fertility preference among adolescents who had exposure to family planning communication was (3.8 children compared to 4.5 children; p < 0.001) among those with no exposure. Furthermore, results show that married adolescents in SSA who had exposure to family planning message had a higher average preferred family size compared to those who were not married (4.8 versus 3.8; p < 0.001).

**Conclusion:**

Exposure to family planning communication has shown the potential to influence adolescents’ fertility preference in sub-Saharan Africa. Adolescents with exposure to family planning messages preferred a small family size. Therefore, there is a need to scale-up family planning education programmes in order to reduce fertility further in SSA.

## Introduction

Population growth remains a major subject of concern among development practitioners, policy makers, demographers, and among other professionals the world over [1, 2]. It is forecasted that the world population will reach 9.73 billion around the mid-century [2]. This growth rate has negative consequences, including food insecurity, environmental degradation, poverty, unemployment, low quality of life, uncontrolled urbanization, climate change and political turmoil [3, 4]. However, it is well-known that population growth and projections are not uniform across sub-regions in SSA [5–8]. Sub-Saharan Africa (SSA) is one region among the developing world where population size has been increasing faster than it can generate resources to support it [2, 5]. Recent statistics show that the average total fertility rate for SSA is 4.8 children per woman compared to an average of 3.8 children per woman for all least developing countries worldwide [5]. Evidence shows that even though fertility transition has begun almost everywhere, the decline has been slower in SSA compared to other parts of the world [8–11].

Sub-Saharan Africa countries are not homogeneous as there are important regional variations in total fertility rate. As of 2015, the total fertility rate in many countries in Eastern and Southern Africa has been declining, whereas the total fertility rate in Western and Central Africa has remained stable at approximately 6 children per woman [1, 12]. Fertility has been predominantly higher in Western and Central Africa due to two major factors. In the first place, there has been a low uptake of family planning (FP), with little improvement over time [1, 13, 14]. Another factor that has an adverse impact on family planning programming is fertility preference, which has been predominantly pro-natal[15].

There is consensus in literature that access to family planning education influences reproductive outcomes such as age at first sex, contraception, age at first birth, teenage pregnancy and fertility preference among women of reproductive age [16–21]. Given the observed high levels of fertility in SSA [6, 8, 22], family planning education campaigns have focused on disseminating messages on benefits of smaller family sizes on both maternal and child health. Demographic evidence shows that there is a positive relationship between fertility levels and fertility preference [6, 7]. So that a decrease in fertility preference is likely to result in a decline in total fertility rate. It is therefore prudent that such campaigns should target changing reproductive behaviors of people, especially adolescents. This is because adolescents’ perceived fertility preference is highly likely to affect on a country’s future fertility course [23–25]. Within SSA region, most studies have been conducted to examine fertility preferences are among all women in the reproductive ages 15–49 years [23, 26–30]. Findings from these show that family planning education, place of residence, wealth status and level of women’ education influenced women’s reproductive behaviour, including a preference for smaller family size and increased contraceptive use [15, 16, 31–34]. Therefore, this study sought to examine fertility preferences of adolescent which under studied in SSA.

There are gaps in the literature regarding fertility preferences among female adolescents in sub-Sahara Africa. First, although family planning communication through media or health facility visit has the power to influence fertility behavior especially among adolescents, there are fewer studies that have focused on the role FP communication plays in influencing adolescents’ fertility preference in SSA. Second, there is missing information on how socio-economic and demographic factors influence adolescents’ fertility preference at regional levels in SSA. Considering that there is a vast variation in socio-cultural norms and value for children across countries in SSA [35], it is important to study determinants of fertility preference in adolescents to inform design of regional family planning strategies to reduce fertility in SSA. Earlier studies on adolescents’ fertility preference in SSA have focused on country level analysis. Even though studies on FP and fertility have shown a positive association in most SSA countries, it remains unclear how family planning communication influence fertility preferences among adolescents in SSA. A holistic understanding of how FP communication influences adolescents’ fertility preference would produce information relevant to inform fertility policy and FP programming to effectively contribute to fertility decline in the region.

In this study, we used data from nationally representative cross-sectional surveys to have a comprehensive understanding of the extent of the association between exposure to family planning communication and fertility preference during adolescence in SSA. The findings of this study could inform strengthening of family planning policy suggestions to further reduce fertility in SSA. The study also sought to establish country-level variations regarding the association between exposure to FP messages and fertility preference among adolescents.

## Methods and data

### Data source

This study used data extracted from the most recent Demographic and Health Survey (DHS) datasets for 28 countries in SSA. DHS surveys were conducted between 2008 and 2018 (Table 1). The DHS program draws national representative samples of households which are usually selected via two-stage stratified cluster sampling technique [36]. All women aged 15–49 years and men 15–59 years who spend a night in the household before the interview date are usually selected for interviews. The interviews are conducted using three main questionnaires namely; household questionnaire, woman questionnaire and men questionnaire. Participants in the DHS surveys included in this study were interviewed by field workers who were well-versed in a wide range of sexuality and family planning and reproductive health topics [36]. DHS data are typically weighted to account for the complexity of survey design and response bias, with the goal of ensuring that the sample is representative of the general population [36, 37].

### Study sample

The analysis samples for this study comprised female adolescents’ aged 15–19 years extracted from each country’s recent DHS. Analytic data came from the women individual recode files (IR dataset) for each country. Country samples included all adolescents who were not declared infecund. This resulted in a pooled sample of 87,950 adolescents included in the analysis. The country-level samples ranged from 1,505 adolescents in South Africa to 8,423 in Nigeria. Furthermore, adolescents who reported non numeric fertility preference were excluded from the analysis. A detailed description of the study samples for each country is presented in Table 1:

### Study measures

#### Outcome variable

The outcome variable of interest in this study is fertility preference. Fertility preference is defined as the percentage of women and men according to their desire for children [36]. The DHS program usually collects information on fertility preference from all interviewed women aged 15–49 years. For this study, we limited the outcome variable of interest to only adolescent girls aged 15–19 years who were not declared infecund. To examine fertility preferences of adolescents, we used the DHS question “If you could choose exactly the number of children to have in your whole life, how many would that be?”. In the DHS, the responses were collected as numeric. To permit the analysis of interest, we classified the fertility preference variable in two levels; at the first level, the outcome variable was classified as a count distribution to facilitate computation of the average preferred number of children among adolescents in SSA and across countries included in the study. In the second level, we classified the outcome as binary, such that a threshold of 3 children or fewer was classified as “0” representing preference of a small family size and adolescents who preferred 4 or more children were classified as “1” representing preference of a large family size. This choice for the cut-off was informed by existing literature on determination of low or high fertility [38–40].

#### Independent variables

Based on the literature review, we identified individual and household level predictors that might be associated with fertility preference of adolescents in SSA. These variables are classified as socio-economic and demographic factors. DHS reference materials and data collection forms were used to identify the independent variables of interest presented here. The main predictor variable for this study was exposure to family planning information. This variable is a composite variable that was constructed by combining 5 DHS related variables which collected information on exposure to family planning messages (that is, exposure to FP messages through radio or television or newspaper or exposed to FP messages at health facility or exposure to FP messages through community health work home visits). This process result into a binary variable which was coded as “1” exposed to FP messages and “0” no exposure to FP messages. Other independent variables included in the study were age of adolescent categorised as (15, 16, 17, 18 and 19); current marital status (categorised as never married, currently married/living with partner and formally married; residence (urban; rural); education (no education, primary, secondary, tertiary); household wealth index (categorised as poor, middle, rich); religion (catholic, protestant, Muslim, other); employment status (categorised as employed, unemployed) and contraceptive use (not using a method, using a method) and visited health facility in the last 12 months (yes, no).

### Statistical analysis

Statistical software Stata SE version 17.0 was used to perform complex survey analysis by taking into account sample weight. Descriptive analysis was performed to summarize the study samples for each country included in the study. Categorical variables were presented using frequencies and percentages while means were computed for continuous data. Cross tabulations were performed to explore the bivariate association between exposure to family planning messages and fertility preference for each country and marital status of adolescents. Furthermore, analysis was conducted to statistically assess mean differences in perceived fertility preference between adolescents who were exposed to family planning messages and those who were not in each country and overall, for SSA. Additionally, multivariable binary logistic regression was conducted to examine the association between individual and household level factors and perceived fertility preference of adolescents in SSA. The choice of this model was informed by the dichotomous distribution of the dependent variable. The regression models were fitted in two steps. In the first model (model I) we only included our main explanatory variable (exposure to FP messages). This was followed by model II where all control variables were entered into the model. On the basis of both models, the odds ratios (AOR) were calculated and presented along with their respective 95% confidence intervals (95% CI). To facilitate analysis of pooled data, sample weights were equalized to give equal weights to each survey included in the analysis.

### Ethical approval

The data analysed in this study is available in the public domain at (https://dhsprogram.com/) Permission to the data was obtained from the DHS IPUMS program. All country DHS datasets for 28 countries in SSA did not contain any identifying information. All DHS country studies were approved by the respective country Ethical Review Boards and the Centers for Disease Control and Prevention (CDC) Atlanta.

## Results

### Average fertility preference among adolescents

**Fig. 1:**
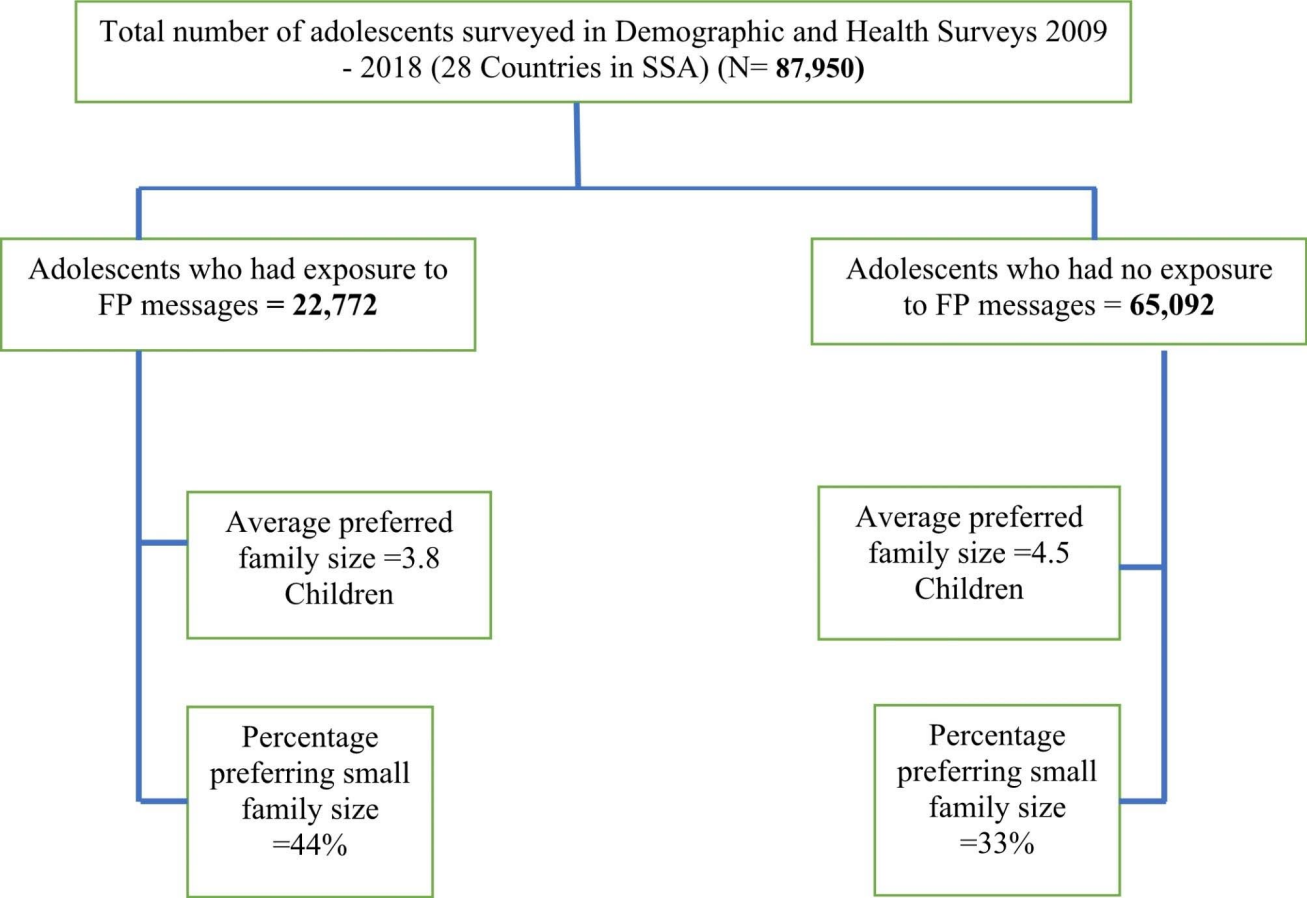
Description of adolescents by exposure to FP messages and fertility preference

Twenty-eight DHS datasets were included in the study. The outline description of the sample information for the study in presented in Table 1 and Fig. 1. Findings show that the average fertility preference among adolescents in SSA was 4.6 (95% CI: 4.5–4.7). The average fertility preference among adolescents ranged from a low of 2.1 children in both Lesotho and South Africa (95% CI: 2.0–2.2) to highs of 9.5 children (95% CI: 8.7–10.4) in Mali and 8.1 (95 CI: 7.9–8.3) in Niger. Furthermore, our study found that, overall, six in every ten adolescents in SSA preferred a large family size. Niger and Chad had the highest proportion of adolescents who preferred at least 4 children (96.0% and 95.6%) respectively, while Lesotho and South Africa had the lowest percentage of adolescents preferring a large family size 7.5% and 11.1% respectively.

**Table 1:** Sample size and descriptive statistics of fertility preference among adolescents across countries in SSA

Country	DHS year	Sample	Average fertility preference (95%CI)	Percentage of adolescents who preferred 4 + children
Angola	2015	3,363	3.9 (3.8, 4.1)	64.3
Benin	2017	3,335	4.6 (4.5, 4.7)	80.1
Burkina Faso	2010	3,349	4.6 (4.6, 4.7)	78.9
Burundi	2016	3,968	3.7 (3.7, 3.8)	50.4
Cameroon	2018	2,676	4.9 (4.7, 5.0)	75.4
Chad	2014	3,705	7.2 (7.1, 7.4)	95.6
Congo DR	2013	3,981	5.3 (5.1, 5.5)	80.3
Cote d’Ivoire	2011	1,997	4.5 (4.4, 4.7)	77.9
Ethiopia	2016	3,498	3.6 (3.4, 3.8)	57.2
Ghana	2014	1,756	3.9 (3.8, 4.0)	60.1
Guinea	2018	2,561	5.0 (4.9, 5.1)	84.6
Kenya	2014	2,862	3.2 (3.1, 3.2)	34.8
Lesotho	2014	1,542	2.1 (2.0, 2.2)	7.5
Liberia	2013	1,915	4.0 (3.8, 4.1)	63.0
Madagascar	2008	4,034	4.1 (3.9, 4.2)	59.1
Malawi	2016	5,273	3.0 (2.9, 3.0)	34.9
Mali	2018	2,209	9.5 (8.7, 10.4)	90.1
Mozambique	2011	3,065	3.8 (3.4, 3.9)	56.3
Namibia	2013	1,857	2.4 (2.4, 2.5)	18.8
Niger	2012	1,901	8.1 (7.9, 8.3)	96.0
Nigeria	2018	8,423	6.8 (6.4, 7.3)	84.3
Rwanda	2014	2,779	3.0 (2.9, 3.0)	23.8
Senegal	2017	3,920	4.9 (4.8, 5.1)	30.0
South Africa	2016	1,505	2.1 (2.0, 2.2)	11.1
Tanzania	2015	2,932	4.1 (4.0, 4.2)	60.1
Uganda	2016	4,276	4.1 (4.0, 4.2)	75.0
Zambia	2018	3,112	3.7 (3.6, 3.8)	60.2
Zimbabwe	2015	2,156	3.3 (3.2, 3.4)	41.7
**Total**		**87,950**	**4.6 (4.5, 4.7)**	**63.8**

Generally, the average fertility preference among adolescents in SSA is highest in countries from Western Africa. Ghana had the lowest proportion of adolescents who had a preference for a large family size in the sub-region (60.1%) while Niger had the highest at 96%. Countries from Southern Africa recorded the least average fertility preference. Lesotho had the lowest percentage of adolescents who preferred a large family size (7.5%) and Angola had the highest at 64.3%.

### Association between exposure to family planning messages and fertility preference

The T-test results for the average fertility preference show that the overall fertility preference among adolescents who had exposure to family planning communication in SSA is significantly lower than the mean fertility preference of those who had no expose to FP communication (3.8 children compared to 4.5 children; (p < 0.001). Generally, in most countries included in this study, the preferred mean fertility preference among adolescents who had exposure to family planning messages was significantly lower compared to those who had no exposure except for Guinea, Lesotho, Namibia and Zambia. Low average fertility preferences among adolescents with exposure to FP communication were observed in Southern and East African countries (Lesotho, South Africa, Namibia and Kenya) while higher average fertility preferences were observed in West African countries (Niger, Chad, Guinea and Mali) (Table 2).

**Table 2:** Distribution of average fertility preference by exposure to family planning messages among adolescents in SSA countries (N = 87,950)

Country	Had exposure to FP messages	Had no exposure to FP Messages	p-value
Angola	3.6	4.0	0.000
Benin	4.1	4.8	0.000
Burkina Faso	4.2	4.9	0.000
Burundi	3.5	3.8	0.000
Cameroon	4.4	5.1	0.000
Chad	6.2	7.4	0.000
Congo DR	4.7	5.4	0.000
Cote d’Ivoire	4.1	4.7	0.000
Ethiopia	3.3	3.7	0.000
Ghana	3.6	4.1	0.000
Guinea	5.2	5.0	0.096
Kenya	2.9	3.4	0.000
Lesotho	2.2	2.1	0.135
Liberia	3.7	4.1	0.000
Madagascar	3.4	4.2	0.000
Malawi	2.9	3.0	0.021
Mali	5.2	5.6	0.006
Mozambique	3.4	3.9	0.000
Namibia	2.4	2.5	0.375
Niger	6.7	8.4	0.000
Nigeria	4.8	5.6	0.000
Rwanda	2.8	3.0	0.005
Senegal	4.2	5.2	0.000
South Africa	2.2	1.9	0.000
Tanzania	3.8	4.4	0.000
Uganda	3.9	4.2	0.000
Zambia	3.8	3.7	0.139
Zimbabwe	3.1	3.4	0.000
**Total**	**3.8**	**4.5**	**0.000**

### Association between marital status and fertility preference among adolescents exposed to family planning messages

Table 3 shows result of the association between marital status and fertility preference among adolescents who were exposed to family planning messages in SSA. The T-test results indicate that the average fertility preference of married adolescents was significantly higher than the mean fertility preference of unmarried adolescents (4.8 children compared to 3.8 children; (p < 0.001). In most countries included in this study the preferred mean fertility preference among married adolescents who had exposure to family planning messages were significantly higher compared to those who were not married except for Angola, Ghana, Kenya, Mali, Namibia, Rwanda, Lesotho, Namibia, Rwanda and South Africa. Low fertility preferences among married adolescents with exposure to FP communication were observed in Southern and East African countries (Lesotho, South Africa, Namibia, Rwanda and Kenya) while higher average fertility preferences among married adolescents were observed in West African countries (Niger = 7.5, Chad = 7.0, Mali = 8.9 and Nigeria = 6.9) (Table 3).

**Table 3:** Distribution of average fertility preference among married and unmarried adolescents who were exposed to family planning messages in SSA countries (N = 87,950)

Country	Married adolescents	Not married adolescents	p-value
Angola	4.0	3.7	0.066
Benin	4.4	4.1	0.025
Burkina Faso	4.9	3.9	0.000
Burundi	3.6	3.4	0.021
Cameroon	4.8	4.2	0.000
Chad	7.0	5.8	0.000
Congo DR	5.9	4.4	0.000
Cote d’Ivoire	4.5	4.0	0.011
Ethiopia	4.6	3.6	0.000
Ghana	3.8	3.6	0.114
Guinea	5.6	5.0	0.011
Kenya	3.2	3.1	0.250
Lesotho	2.5	2.1	0.029
Liberia	4.2	3.8	0.001
Madagascar	4.1	3.1	0.000
Malawi	3.1	2.8	0.000
Mali	8.9	7.9	0.359
Mozambique	3.7	3.1	0.000
Namibia	2.2	2.3	0.403
Niger	7.5	6.0	0.000
Nigeria	6.9	5.5	0.002
Rwanda	2.8	2.9	0.999
Senegal	5.8	4.4	0.000
South Africa	2.4	2.2	0.369
Tanzania	4.6	3.7	0.000
Uganda	4.2	3.9	0.000
Zambia	4.2	3.8	0.001
Zimbabwe	3.6	2.9	0.000
**Total**	**4.8**	**3.8**	**0.000**

### Determinants of preference for a large family size among adolescents in SSA

Multivariable logistic regression was used to examine the influence of explanatory variables on fertility preference among adolescents in SSA. Results for model I show that exposure to family planning messages is associated with reduced likelihood of preferring a large family size by 42% in adolescence (Table 4). In the full model, most independent variables used in the study, that is age, marital status, education level, employment status, visiting health facility in the last 12 months and exposure to FP messages, were significantly associated with adolescents’ fertility preference. Results show that adolescents aged 19 years (AOR = 0.93, 95% CI: 0.87–0.99) were less likely to prefer a large family size. Those in marital union (AOR = 2.02, 95% CI: 1.89–2.17) were more likely to prefer a large family. Education level of adolescents was negatively associated with fertility preference. An increase in education level was associated with reduced odds of preferring a large family size (AOR = 0.22, 95% CI: 0.18–0.28) for adolescents with higher level of education and (AOR = 0.26, 95% CI: 0.24–0.29) for adolescents with secondary level of education. Furthermore, results in Table 4 show that visiting the health facility in the last 12 months (AOR = 0.81, 95% CI: 0.77–0.84) was associated with reduced odds of preference for a large family size.

**Table 4:** Results of multivariable regression analyses examining the effect of individual level factors on fertility preference among adolescents in SSA (N = 87,950)

Background Characteristics	Model I		Model II	
	OR	95% Confidence Internal	AOR	95% Confidence Internal
**Exposure to FP messages**				
No	Ref		Ref	
Yes	0.58***	(0 0.55–0.61)	0.76***	(0.72–0.80)
**Age**				
15			Ref	
16			1.01	(0.95–1.07)
17			1.03	(0.97–1.10)
18			1.02	(0.96–1.08)
19			0.93*	(0.87–0.99)
**Residence**				
**Urban**			Ref	
Rural			1.00	(0.94–1.06)
**Marital status**				
Never married			Ref	
Married			2.02***	(1.89–2.17)
Formerly married			1.09	(0.93–1.26)
**Working status**				
No			Ref	
Yes			1.31***	(1.24–1.36)
**Children ever born**				
Zero			Ref	
One or more			1.00	(0.94–1.07)
**Education level**				
None			Ref	
Primary			0.28***	(0.26–0.30)
Secondary			0.26***	(0.24–0.29)
Higher			0.22***	(0.18–0.28)
**Household wealth status**				
Poor			Ref	
Moderate			0.81***	(0.77–0.87)
Rich			056***	(0.18–0.59)
**Visited health facility in the last 12 months**				
No			Ref	
Yes			0.81***	(0.77–0.84)

*** *p* < 0.001; ** = *p* < 0.01; * = *p* < 0.05;

## Discussion

The study focused on examining the influence of exposure to family planning messages on fertility preference among adolescent girls in SSA. Study also examined country variations in the prevalence of desire for a large family size among adolescents. Our review of the literature reveals that there has been no known comprehensive study of this nature conducted before in SSA and thus bolstering the importance of our findings. We focused on understanding adolescents’ fertility preference because the future fertility of a country is highly likely to be influenced by present adolescents’ reproductive behavior. Since family planning services are mostly offered during antenatal and under five clinics, adolescents may prefer getting family planning information via mass-media channels [41]. Our study revealed that exposure to family planning messages was significantly associated with adolescents’ family size preference in SSA. Other variables found to be important included age, marital status, household wealth status, education level, and visiting the health facility in the last 12 months prior to data collection.

Study findings revealed that adolescents who had exposure to family planning messages were 24% less likely to preference a large family size comparable to those who had no exposure. This can be attributed to the appreciation of benefits of family planning education gained by adolescents through mass-media, visit to health facility and community visits by community health workers. Family planning messages are usually targeted at influencing individuals’ reproductive behavior towards contraception, limiting of births, spacing of children and choice of small family. This finding implied a significant contribution of family planning education interventions to social and reproductive behavior change among adolescents in SSA. Similar results were reported in a study conducted in Rwanda in 2016 [42] where reduction in fertility preference among women of reproductive age was attributed to massive family planning education through mass-media and community-led sensitization programmes.

The study found that adolescents who were married or living with a partner were twice as likely to prefer a large family size compared to the never married. In most African culture, we may expect women to have children soon after marrying, causing partnered women to stop using contraception [43]. Such practices could explain why teenage pregnancy and early motherhood are high in SSA. There may also be sub-regional disparities in the norms surround timing of having a child after marriage. This result implies the urgent need for community led family planning education programmes aimed at influencing reproductive behavior change of married adolescents, especially those coming from rural settings.

Earlier studies have shown that education and wealth status are strongly associated with fertility preference [9, 44, 45] such that individuals with higher level of education and those from higher wealth groups have a tendency to preference a low family size. This is because this demographic group is expected to have adequate information about benefits that accrue with smaller family sizes. Our study confirms the findings presented by earlier studies. We established that adolescents with secondary or higher-level education were 74% and 78% less likely to preference a large family size, respectively. These findings are consistent with similar studies conducted in Zambia, Rwanda, Burkina Faso, Niger, Mali and Egypt that reported education and wealth status as significant predictors of fertility preference [44, 46–48]. This implies that education is an important component for reducing fertility in SSA. Therefore, education policies should propagate the implementation of strategies that improve education access to girls and young women, especially those in marginalized communities.

Access to family planning communication is another issue that needs to be addressed in most countries in SSA. Places such as schools, community youth friendly corners, private pharmacies/drug stores and traditional ceremonies can also serve as distribution points for family planning information to adolescents. The merits of disseminating family planning information in schools through the introduction of comprehensive sexuality education need to be explored further. Improving the demand for family planning information among adolescents should be stressed in the country’s population policies as a key priority strategy to reduce fertility further. Increasing access to family planning information is essential and has shown to have a significant impact on decision making to use contraception, postponing of marriage and limiting the number of children, thus reducing fertility [49, 50]. Health education on limiting family size through family planning programming will surely help in changing reproductive behavior of adolescents, but it will only be effective if adolescents will embrace the advantages of having smaller families.

Although the study has provided useful findings to inform strengthening of family planning education programmes targeting at changing adolescents’ reproductive behavior. There are a few limitations that could make the conclusions from the study to be interpreted with caution. First, because our data is cross-sectional, we cannot conduct causality analyses, which limits our ability to understand the complexities of adolescents’ experiences regarding their preference through their life cycle. Our study did not examine the influence of community level factors, which are equally important in understanding adolescents’ fertility behavior. As a result, our findings highlight the need for additional research, particularly qualitative and longitudinal research, to further the understanding of the complex interplay between the various individual and community factors that shape adolescents’ reproductive behavior and preference for children, as well as how these factors change over the course of their lives. Finally, because of the lack of other country level data on the IPUMS DHS website, the study did not utilize data for all countries in SSA. Furthermore, IPUMS DHS has not yet updated data for some countries that have conducted new DHS. As a result, the conclusions of this study should not be extrapolated beyond the sub-sample of countries included in our analysis.

## Conclusion

This study has shown that family exposure to family planning messages has the potential to influence adolescents’ fertility preference in SSA. The study has established that the factors associated with fertility preference among adolescent in SSA include marital status, education level, and exposure to family planning messages. There is a need for governments and stakeholders, especially in countries with high fertility levels, to consider strengthening of family planning communication programmes as a top priority targeting mostly in and out-of-school adolescents in order to reduce fertility further in the region. It is important also to consider incorporating sexual reproductive health education into early primary and secondary level curriculum to maximize benefits of family planning programmes. Further research is needed to examine how exposure to family planning messages operates through community level factors to influence fertility preference among adolescents across different countries in SSA. Future research should examine whether socio-economic and cultural factors influence fertility preference differently in married and unmarried adolescents.

## Data Availability

Data used in our study is publicly available upon request from DHS program website. (https://dhsprogram.com/).

## List of abbreviations

CI: Confidence Interval
CPR: Contraceptive prevalence rate
DHS: Demographic and Health Survey
EA: Enumeration Area
FP: Family Planning
SDG: Sustainable Development Goal
SRH: Sexual Reproductive Health
SRHR: Sexual Reproductive Health Rights
SSA: SSA
UNFPA: United Nations Population Fund
USAID: United States Aid for International Development
WHO: World Health Organisation
